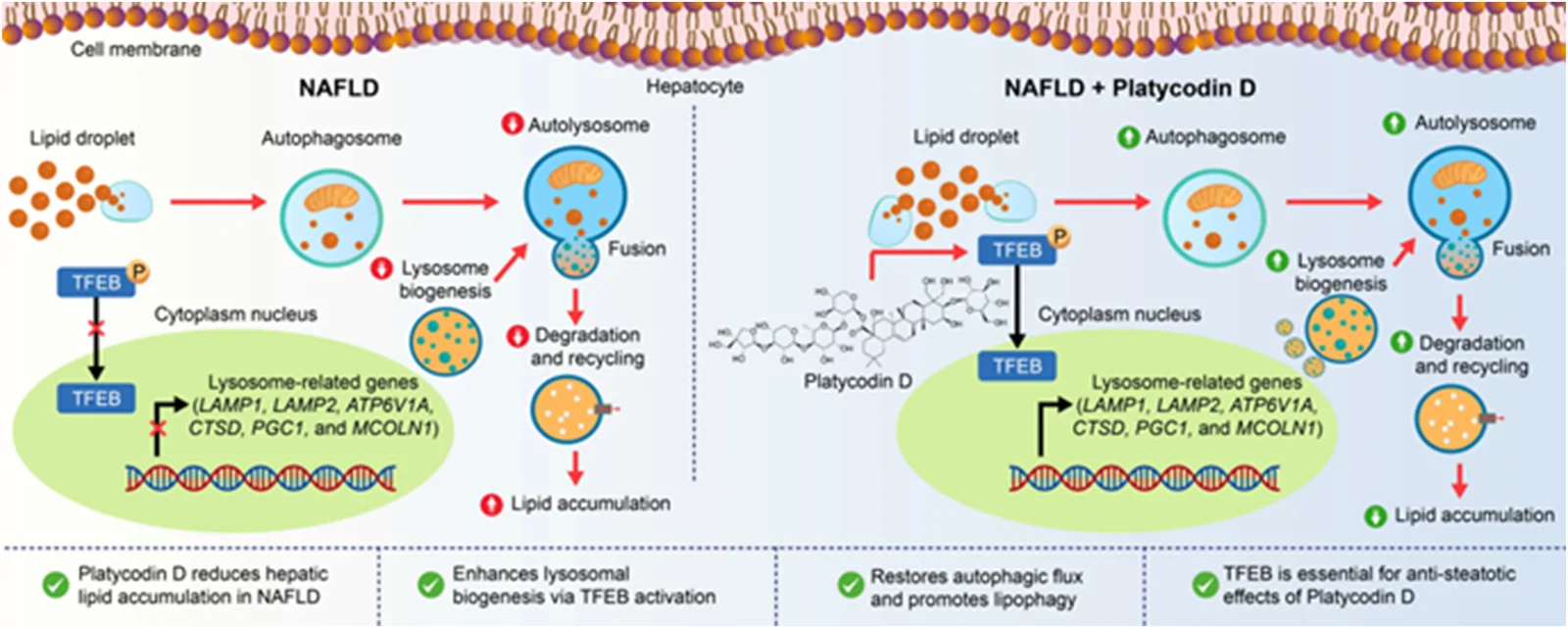# Transcription factor EB activation by platycodin D: A novel therapeutic strategy for hepatic steatosis through enhanced lipophagy

**DOI:** 10.1016/j.gendis.2026.102149

**Published:** 2026-03-18

**Authors:** Soo Yeon Kim, Siwoo Lee, Hee-Jeong Jin

**Affiliations:** a Korean Medicine (KM) Data Division, Korea Institute of Oriental Medicine, Daejeon 34054, Republic of Korea; b Department of Sasang Constitutional Medicine, Gachon University, Seongnam 13120, Republic of Korea

Hepatic steatosis, a hallmark of nonalcoholic fatty liver disease (NAFLD), is characterized by abnormal accumulation of lipids within hepatocytes.^1^ Lipophagy mediates the autophagic degradation of lipid droplets and limits intracellular lipid deposition,^2^ thereby contributing to the prevention of steatosis. Notably, defective lipophagy is a pathological hallmark of NAFLD,^2^ driving its progression. The aim of this study was to investigate the therapeutic potential of platycodin D, a saponin compound derived from the roots of *Platycodon grandiflorus*, in NAFLD and to elucidate the mechanisms underlying its action against hepatic steatosis. Platycodin D treatment reduced lipid droplet accumulation in palmitate-induced hepatocytes; enhanced lysosomal biogenesis through up-regulation of *LAMP1*, *LAMP2*, *ATP6V1A*, *CTSD*, *PGC1*, and *MCOLN1*; and restored impaired autophagic flux, thereby promoting lipophagy. Mechanistically, platycodin D activated the nuclear translocation of transcription factor EB (TFEB), a master regulator of lysosomal biogenesis. Importantly, TFEB deficiency abolished platycodin D-mediated lysosomal biogenesis, autophagic flux, lipophagy, and lipid clearance. Collectively, these findings demonstrate that platycodin D exerts potent anti-steatotic effects by activating TFEB-dependent lysosomal biogenesis and lipophagy, suggesting its potential as a therapeutic agent for NAFLD.

We investigated the influence of platycodin D (Fig. 1A) on hepatic steatosis using palmitate-treated hepatocytes. Treatment with 20 μM platycodin D and 0.2 mM palmitate did not significantly affect cell viability (Fig. S1A and S1B). Bodipy 493/503 staining revealed that platycodin D markedly reduced palmitate-induced lipid droplet accumulation (Fig. 1B; Fig. S2A). Consistently, intracellular triglyceride and total cholesterol levels were significantly decreased following platycodin D treatment (Fig. 1C and D). These results indicate that platycodin D alleviates palmitate-induced lipid accumulation in hepatocytes. Next, we examined whether platycodin D-induced autophagy affects lipid deposition. Hepatocytes were treated with palmitate to mimic steatosis. Palmitate exposure increased LC3 puncta (autophagy marker) and p62 (autophagy substrate) levels, indicating impaired autophagic flux, likely due to disrupted autophagosome-lysosome fusion (Fig. 1E; Fig. S2B and S2C). Subsequent treatment with platycodin D further enhanced LC3 puncta while reducing p62, suggesting restoration of autophagic flux and accelerated clearance of autophagic substrates (Fig. 1E; Fig. S2B and S2C). Using mCherry-GFP-LC3 transfection, platycodin D increased red puncta representing autolysosomes, confirming enhanced autophagosome–lysosome fusion (Fig. 1F; Fig. S2D). Consistently, Bodipy 493/503 staining showed reduced lipid accumulation with platycodin D, an effect reversed by chloroquine (Fig. 1G; Fig. S2E–S2G), indicating that platycodin D reduces palmitate-induced hepatic steatosis by promoting lipophagy through restoration of autophagic flux. Given that enhanced lysosome biogenesis can restore autophagic flux impaired by lipid accumulation,^3^ we examined whether platycodin D regulates lysosome biogenesis. RT-PCR analysis revealed that lysosome biogenesis-related genes, including *LAMP1*, *LAMP2*, *ATP6V1A*, *CTSD*, *PGC1*, and *MCOLN1*, were significantly up-regulated following platycodin D treatment (Fig. 1H). It also promoted colocalization of LC3 with LAMP2, indicating improved autophagosome–lysosome fusion (Fig. 1I; Fig. S3A). Furthermore, the colocalization of lipid droplets with lysosomes was increased in platycodin D-treated cells, suggesting enhanced lipophagy (Fig. 1J; Fig. S3B). These findings demonstrate that platycodin D alleviates lipid accumulation by stimulating lysosome biogenesis and restoring autophagic flux. To evaluate how platycodin D influences TFEB activation, we investigated its effect on TFEB nuclear translocation and the transcriptional regulation of its target genes in hepatic cells. Immunofluorescence staining data showed that platycodin D significantly increased the nuclear translocation of active TFEB in hepatocytes compared with palmitate-induced hepatocytes (Fig. 1K; Fig. S4A). To validate the role of TFEB in regulating lysosome-associated genes, we performed transient silencing of TFEB in hepatocytes. TFEB silencing markedly reduced the mRNA expression of key lysosomal genes, such as *LAMP1*, *LAMP2*, *ATP6V1A*, *CTSD*, *PGC1*, and *MCOLN1* (Fig. 1L), and its knockdown attenuated red (mCherry; acid-stable) puncta in platycodin D-stimulated hepatocytes (Fig. 1M; Fig. S4B). TFEB knockdown significantly impaired the colocalization of autophagosomes and lysosomes in platycodin D-treated hepatocytes, as evidenced by reduced colocalization in fluorescence staining (Fig. 1N; Fig. S4C). Furthermore, the colocalization of lipid droplets and lysosomes by staining with Bodipy 493/503 and LysoTracker significantly was decreased in platycodin D-treated hepatocytes following TFEB knockdown (Fig. 1O; Fig. S4D). These findings suggest that platycodin D activates TFEB by promoting lysosomal biogenesis, thereby enhancing autophagic flux and facilitating lipid clearance in hepatic cells.

**Figure 1:**
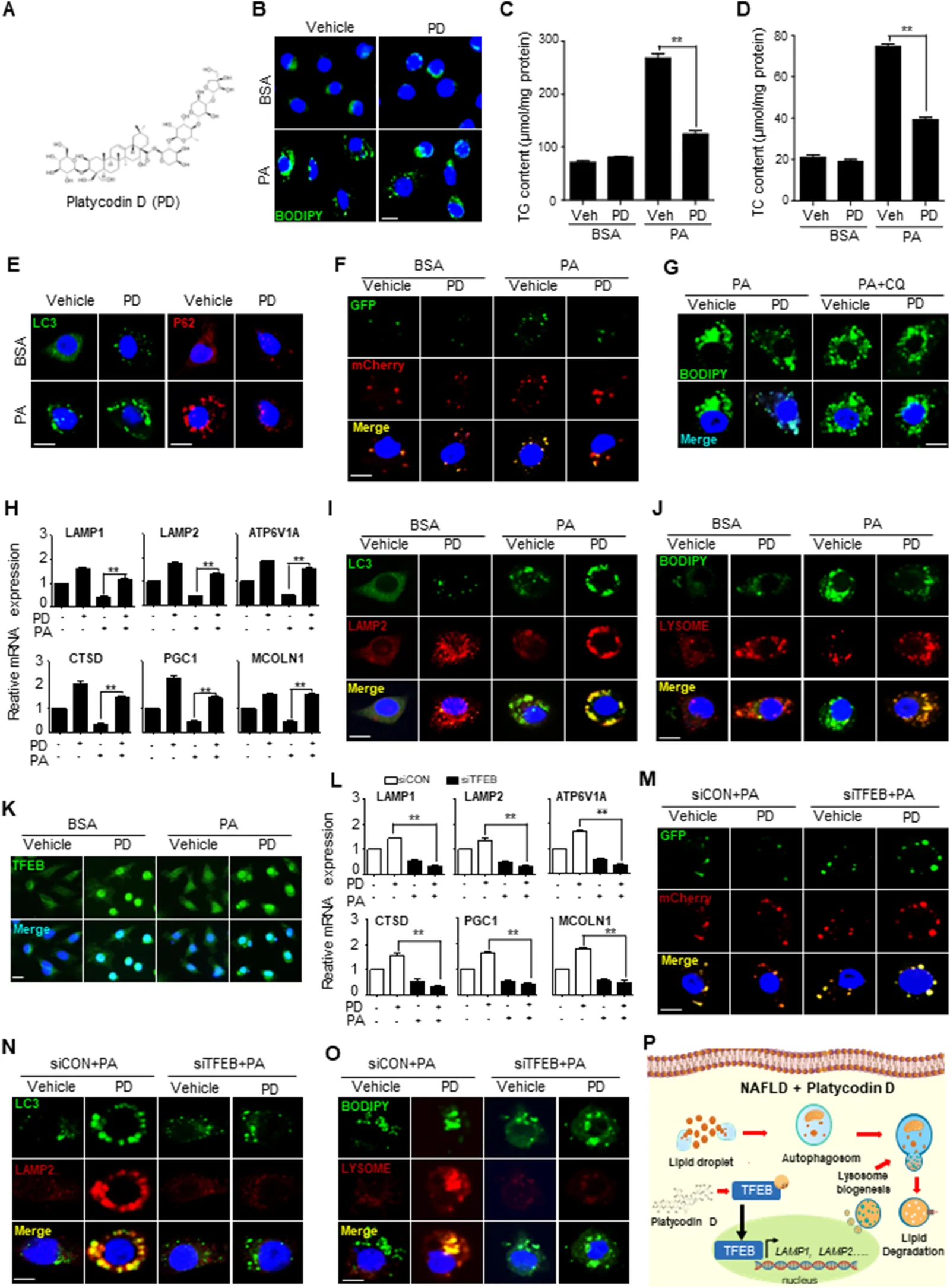
Platycodin D alleviates hepatic steatosis by promoting transcription factor EB (TFEB)-mediated lysosomal biogenesis, restoring autophagic flux, and enhancing lipophagy. **(A)** The chemical structure of platycodin D. (B–O) NCTC clone 1469 cells were stimulated with or without palmitate (0.2 mM) in the presence and absence of platycodin D (20 μM). **(B)** NCTC clone 1469 cells were stained with BODIPY 493/503 (green). Merged signals of BODIPY 493/503 and DAPI (nuclei, blue). **(C)** Intracellular triglycerides were measured using a quantification kit. **(D)** Intracellular total cholesterols were measured using a quantification kit. **(E)** Immunofluorescence microscopy analysis of LC3 puncta (green) or p62 (red). Merged signals of LC3 (Alexa Fluor 488-conjugated goat anti-rabbit IgG, green) and DAPI (nuclei, blue). Merged signals of p62 (Alexa Fluor 594-conjugated goat anti-mouse IgG, red) and DAPI (nuclei, blue). **(F)** NCTC clone 1469 cells were transfected with the LC3B plasmid (mCherry-EGFP-LC3B) for 24 h. Representative immunofluorescence images. **(G)** NCTC clone 1469 cells were stained with BODIPY 493/503 in the presence and absence of CQ (20 μM). **(H)** LAMP1, LAMP2, ATP6V1A, CTSD, PGC1 and MCOLN1 mRNA expression using quantitative real-time reverse transcription-polymerase chain reaction (RT-PCR). **(I)** Immunofluorescence microscopy analysis of a merge of LC3 puncta (green) and LAMP2 (red) staining. **(J)** NCTC clone 1469 cells were stained with BODIPY 493/503 (green) and LysoTracker (red). Immunofluorescence microscopy analysis of a merge of BODIPY 493/503 and LysoTracker. **(K)** TFEB localization was determined by confocal fluorescence microscopy. (L–O) NCTC clone 1469 cells were transfected with non-silencing control siRNA (control) or siRNA targeting *TFEB* (siTFEB). **(L)** LAMP1, LAMP2, ATP6V1A, CTSD, PGC1 and MCOLN1 mRNA expression using RT-PCR. **(M)** NCTC clone 1469 cells were transfected with the LC3B plasmid (mCherry-EGFP-LC3B) for 24 h. Representative immunofluorescence images. **(N)** Immunofluorescence microscopy analysis of a merge of LC3 puncta and LAMP2 (red) staining. **(O)** NCTC clone 1469 cells were stained with BODIPY 493/503 (green) and LysoTracker (red). Immunofluorescence microscopy analysis of a merge of BODIPY 493/503 and LysoTracker. **(P)** Working model illustrating how platycodin D alleviates hepatic steatosis by activating TFEB-mediated lysosomal biogenesis, restoring autophagic flux, and enhancing lipophagy. Data are expressed as the mean ± SD. ∗∗*P* < 0.01. Scale bar: 10 μm. Veh, vehicle; PA, palmitate; PD, platycodin D.

The present study demonstrates that platycodin D effectively mitigates palmitate-induced lipid droplet accumulation by stimulating lysosomal biogenesis and restoring impaired autophagic flux. This process is driven by the robust transcriptional up-regulation of lysosome-associated genes, which subsequently facilitates lipophagic clearance of hepatic lipids. TFEB is a key regulator of lysosomal biogenesis and autophagy, and its dysregulation impairs autophagic flux, leading to hepatic lipid accumulation and NAFLD progression.^4^ Pharmacological activation of TFEB restores lysosomal function and lipophagy, highlighting its therapeutic potential in NAFLD.^5^ The novelty of platycodin D as a TFEB activator is underscored by its ability to modulate MCOLN1 at the transcriptional level, a mechanism that distinguishes it from other phytochemicals. Our data suggest that platycodin D-induced up-regulation of MCOLN1 facilitates lysosomal calcium efflux via TRPML1 channels, subsequently triggering calcineurin-dependent TFEB dephosphorylation and its accelerated nuclear translocation. Notably, this robust TFEB activation significantly enhances lysosomal biogenesis and the rapid restoration of lysosomal integrity, leading to an autophagic flux efficiency that appears far superior to that of previously reported natural compounds. Furthermore, the amphiphilic moiety of platycodin D facilitates superior membrane partitioning and efficient cellular internalization, thereby eliciting an augmented TFEB-mediated response at a higher efficiency than previously documented phytochemicals. In this study, a key finding was that platycodin D activates TFEB and promotes its nuclear translocation, a critical step for initiating lysosomal biogenesis. Together, platycodin D enhanced TFEB-mediated transcription of genes participating in lysosomal biogenesis and autophagy. Importantly, TFEB knockdown suppressed the expression of lysosome-associated genes and impaired autophagic flux and lipid clearance, abolishing the lipophagy-enhancing effects of platycodin D. These observations highlight the essential role of TFEB as a central mediator of the anti-steatotic effects of platycodin D. Our findings are in agreement with previous findings demonstrating that TFEB activation restores lysosomal biogenesis and autophagic degradation of lipid droplets, ultimately improving hepatic lipid metabolism.

Despite these promising results, this study has limitations that warrant further investigation. First, future investigations using high-fat diet-induced NAFLD mouse models are essential to validate the translational efficacy of platycodin D within a complex physiological environment. Furthermore, utilizing a liver-specific TFEB knockout (L-TFEB KO) system via Cre-LoxP technology will definitively determine whether the hepatoprotective effects of platycodin D are intrinsically driven by hepatic TFEB signaling or result from systemic off-target actions. Such studies are crucial for establishing platycodin D as a viable TFEB-targeted intervention for metabolic liver diseases. Second, while the exact upstream regulators of platycodin D-mediated TFEB activation remain to be fully elucidated, the induction of the lysosomal calcium–calcineurin axis represents a highly plausible mechanism. Given the known capacity of triterpenoid saponins to perturb calcium homeostasis, platycodin D may trigger lysosomal calcium efflux via TRPML1 (MCOLN1) channels, promoting calcineurin-dependent TFEB dephosphorylation and nuclear translocation. We intend to verify this hypothesis using pharmacological inhibitors, such as BAPTA-AM and cyclosporin A, combined with real-time calcium imaging, to definitively map the TRPML1–calcineurin–TFEB signaling cascade.

In conclusion, platycodin D effectively alleviates hepatic steatosis by restoring impaired autophagic flux through TFEB-mediated activation of lysosomal biogenesis in hepatocytes (Fig. 1P; Fig. S5). To our knowledge, this is the first report to demonstrate that platycodin D alleviates hepatic steatosis via TFEB-dependent autophagic flux enhancement. These findings highlight the translational potential of platycodin D in mitigating metabolic liver disorders driven by defective lipophagy.

## CRediT authorship contribution statement

**Soo Yeon Kim:** Writing – review & editing, Writing – original draft, Validation, Investigation, Formal analysis, Conceptualization. **Siwoo Lee:** Resources. **Hee-Jeong Jin:** Writing – review & editing, Writing – original draft, Project administration, Methodology, Investigation, Funding acquisition, Formal analysis, Conceptualization.

## Funding

This study was supported by the Development of Korean Medicine Original Technology for Preventive Treatment based on Integrative Big Data and Development of systems KM modeling technology connecting Qi-blood circulation and the organ function grant from the 10.13039/501100003718Korea Institute of Oriental Medicine (No. KSN1732121, KSN25120212).

## Conflict of interests

The authors declare no competing interests.

## Figures and Tables

**figs1:**
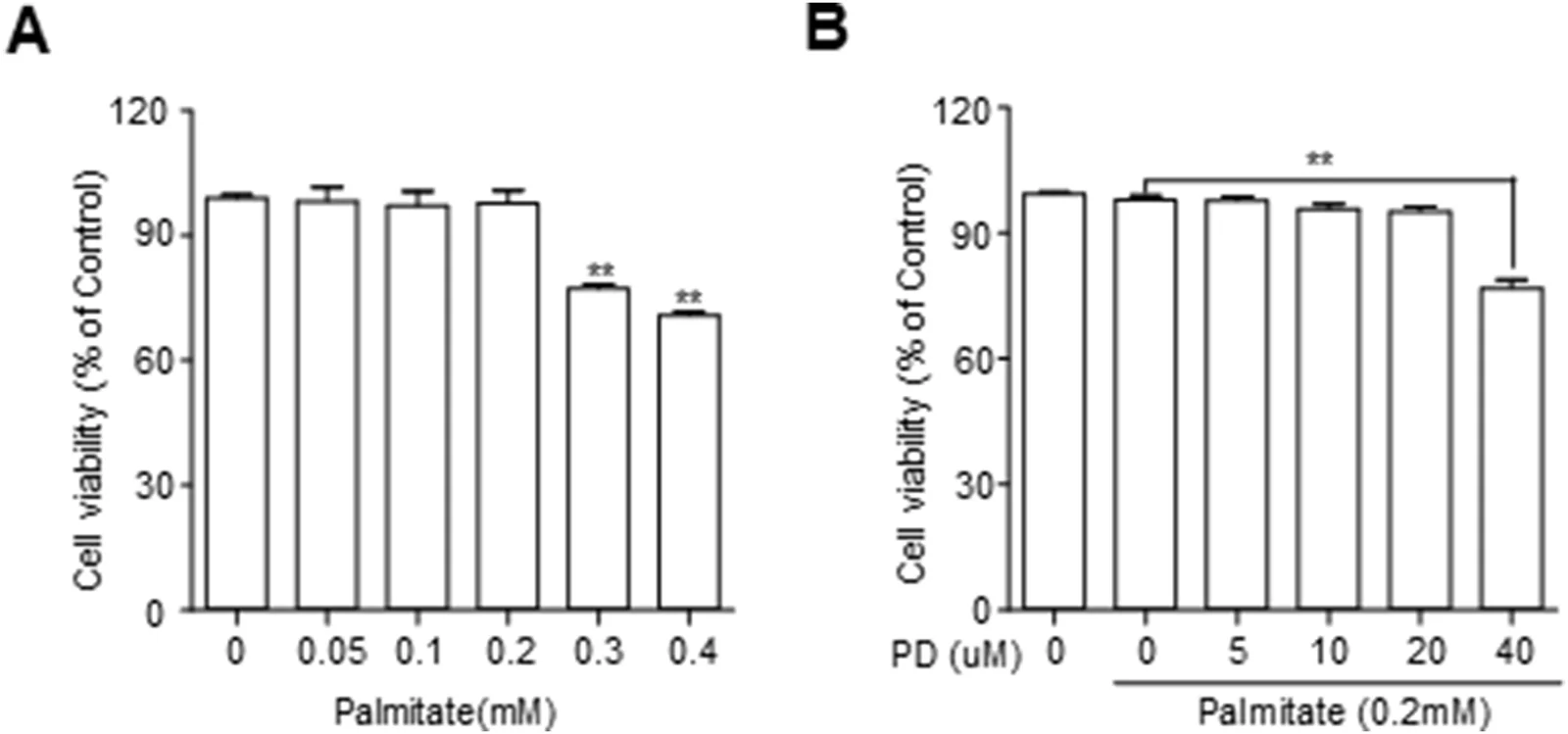


**figs2:**
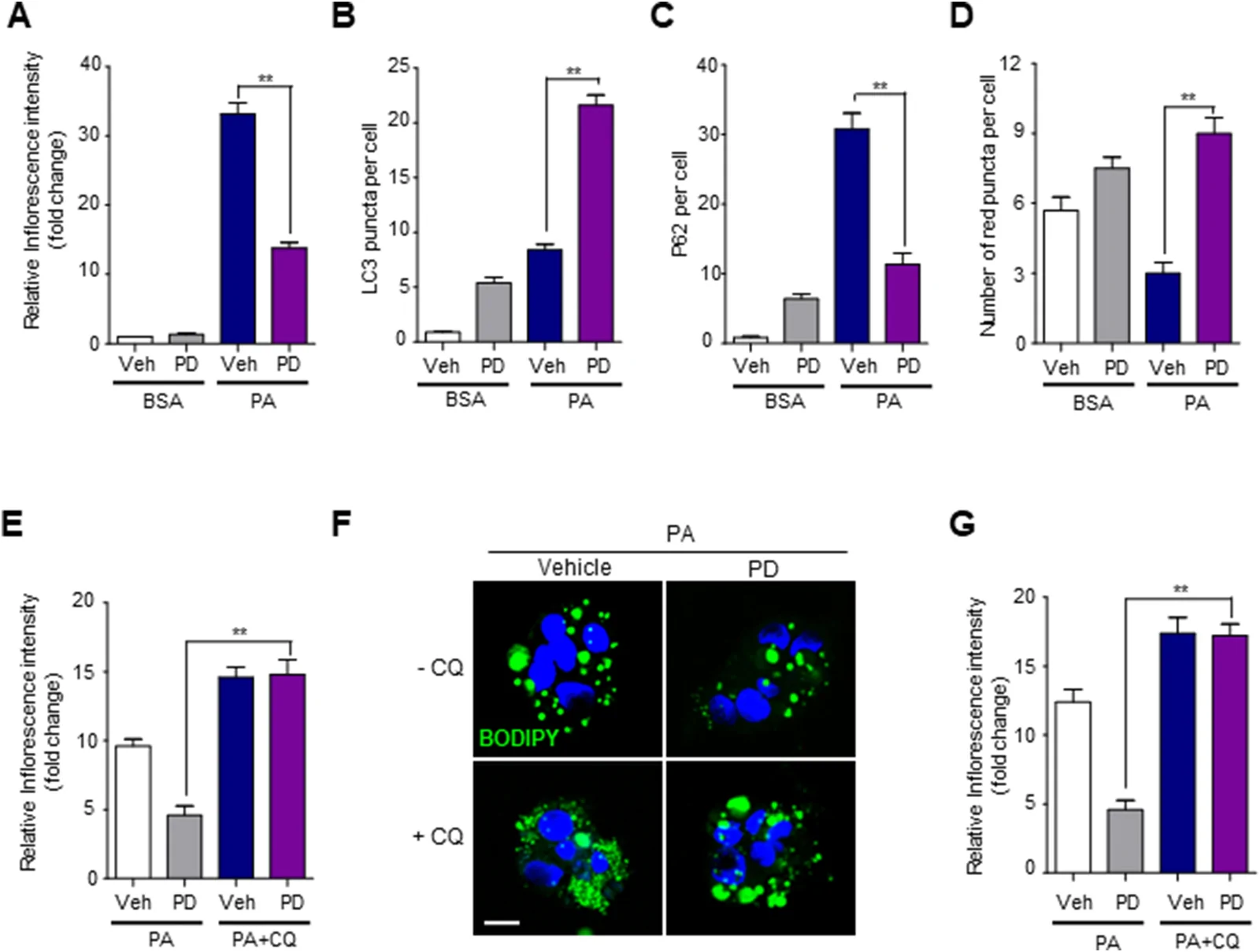


**figs3:**
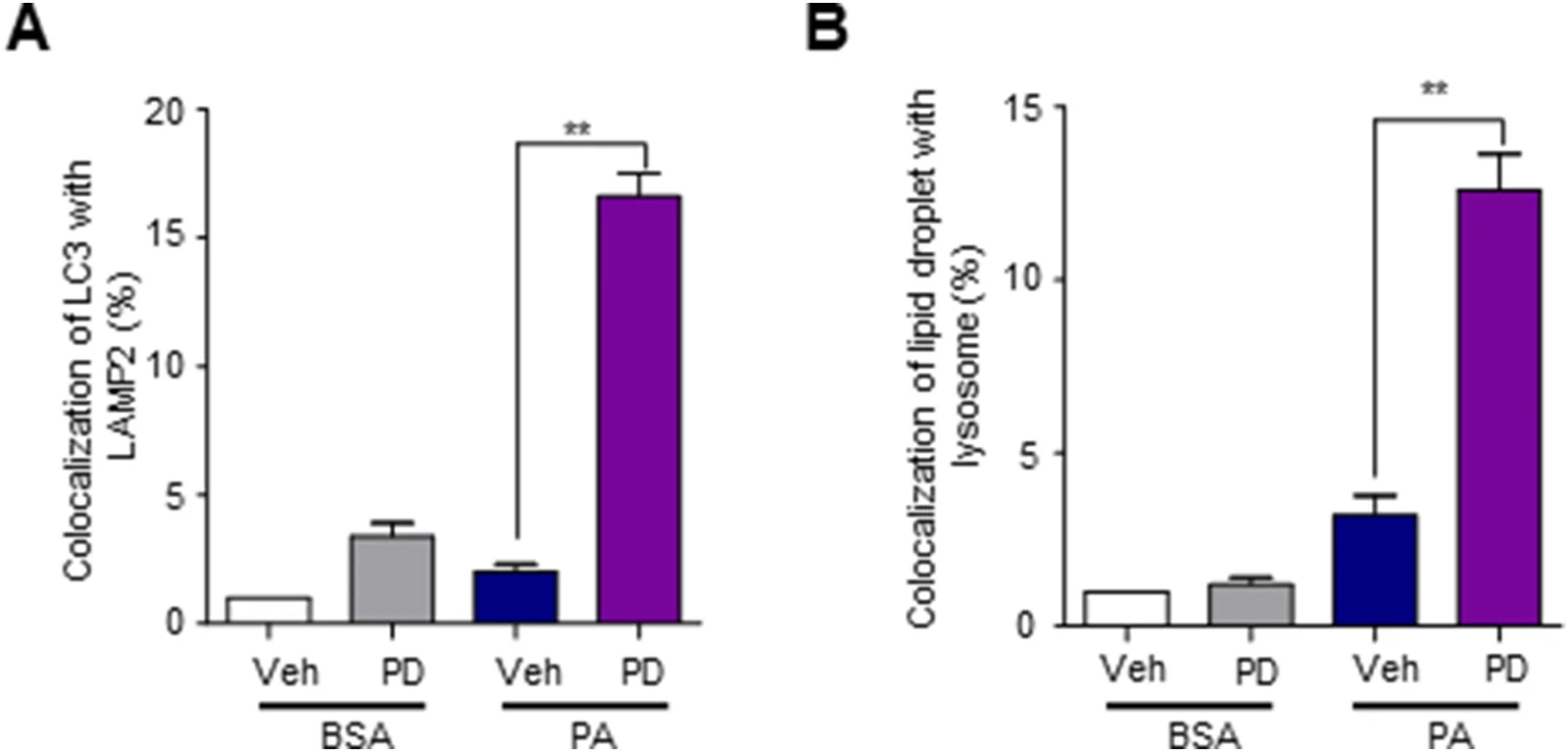


**figs4:**
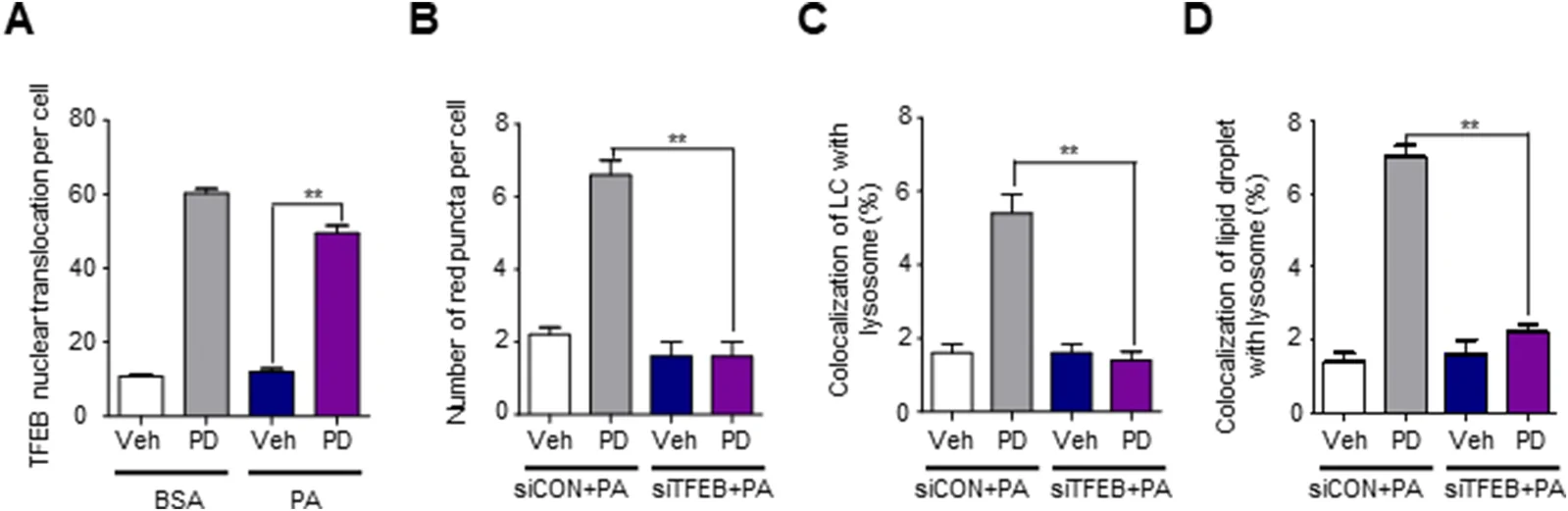


**figs5:**