# Supplementary material for: Transcription factor EB activation by platycodin D: A novel therapeutic strategy for hepatic steatosis through enhanced lipophagy
Source: Genes Dis. 2026 Mar 18;14(2):102149. doi: 10.1016/j.gendis.2026.102149 (PMC13651672; doi:10.1016/j.gendis.2026.102149)
Supplement: Supplementary file 1 [file mmc1.docx]

**Supplementary Information**

This file includes:

Supplementary Background

Supplementary Materials and Methods

Supplementary References

Supplementary Figure Captions (S1-5)

**Supplementary Background**

Nonalcoholic fatty liver disease (NAFLD), the most prevalent chronic liver disease worldwide^1^, affects an estimated 10%–30% of the population and is gradually increasing in incidence^2^. It is characterized by excessive accumulation of lipids in liver cells independent of alcohol consumption and ranges from simple steatosis to nonalcoholic steatohepatitis, which leads to fibrosis and subsequent cirrhosis^3^. NAFLD is closely related to metabolic syndrome conditions, including obesity, dyslipidemia, hypertension, and insulin resistance^4,5^.

Lipophagy regulates lipid metabolism homeostasis by facilitating the lysosome-driven degradation pathway for lipids, regulated by autophagy-related mechanisms. It represents a selective subtype of autophagy in which lipid droplets are recognized and sequestered by autophagosomes, subsequently fusing with lysosomes and leading to the degradation of lipid components^6^. In NAFLD, lipophagy is impaired and lipase-mediated lipid hydrolysis is dysregulated in hepatocytes^7,8^. Notably, disrupted lipophagy is a key hallmark of pathology of NAFLD^8-10^, highlighting a significant correlation between hepatocyte lipophagy and the progression of NAFLD.

Transcription Factor EB (TFEB) plays a pivotal role in maintaining cellular homeostasis by coordinating lysosomal biogenesis and autophagy, including lipophagy^11,12^. As a master transcriptional regulator, TFEB controls the expression of genes involved in lysosomal function and autophagosome formation by binding to CLEAR (Coordinated Lysosomal Expression and Regulation) elements^11,12^. Recent studies have shown that impaired TFEB activity contributes to defective lipophagy and hepatic lipid accumulation, thereby promoting the progression of NAFLD^13-16^. Conversely, pharmacological activation of TFEB enhances lysosomal degradative capacity, restores lipid homeostasis, and alleviates hepatic steatosis^14-16^. Thus, targeting TFEB activation has emerged as a promising therapeutic strategy for NAFLD and other lysosomal storage or metabolic diseases.

*Platycodon grandiflorus*, a member of the family Campanulaceae, has traditionally been used in Asia to provide therapeutic benefit in respiratory diseases, such as sore throat, wheezing, and cough with phlegm^17,18^. Among its bioactive compounds, platycodin D, extracted from its roots, exhibits various biological and pharmacological properties, including anti-inflammatory^19^, anti-tumor^20^, antioxidant^21^, metabolic regulatory^22^, antiviral^23^, and hepatoprotective effects^24^. Previous studies have shown that platycodin D exerts multifaceted metabolic effects. It inhibits intestinal fat absorption, thereby alleviating obesity and NAFLD induced by a high-fat diet (HFD)^25^. Additionally, platycodin D improves hyperglycemia and liver metabolic dysfunction in type 2 diabetic mice induced by HFD and streptozotocin^22^. However, data on the actions of platycodin D in NAFLD remain limited.

**Supplementary Materials and Methods**

**Cell culture**

NCTC clone 1469 cells were obtained from the Korea Cell Line Bank (Seoul, Korea) and maintained in Dulbecco’s modified Eagle’s medium (DMEM; Lonza) supplemented with 10% fetal bovine serum (FBS) and 1% antibiotic–antimycotic solution. HepG2 cells (HB-8065), obtained from the American Type Culture Collection (Manassas, VA, USA), were cultured under identical conditions at 37°C in a humidified 5% CO₂ incubator.

**Cell viability assay**

NCTC clone 1469 cells (1 × 10^3^ /well) were seeded and cultured in a 96-well plate at 37°C and 5% CO_2_ for 24 h. Cells were then treated with or without palmitate (0.2 mM) for additional 24 h and in the presence or absence of platycodin D (20 μM) (SMB00424, Sigma-Aldrich) (Fig. 1A). Cells were incubated with 10 μl of CCK-8 solution in each well for 1 h. A microplate reader was used to measure the absorbance at 450 nm.

**Real-time quantitative polymerase chain reaction**

Total RNA was extracted using TRIzol reagent (15596026, Invitrogen) according to the manufacturer’s instructions. Complementary DNA (cDNA) was synthesized from 1 µg of total RNA using the SuperScript First-Strand Synthesis System (11904018, Invitrogen). Quantitative real-time PCR (qRT-PCR) was performed using SYBR Green PCR Master Mix (4367659, Applied Biosystems) on a Rotor-Gene Q 2plex system (Model 9001620, Qiagen, Hilden, Germany). The thermal cycling conditions consisted of an initial denaturation at 95°C for 10 min, followed by 40 cycles of 95°C for 15 s and 55°C for 60 s. Gene-specific primers were used for amplification, and all reactions were conducted in technical triplicate. Relative mRNA expression levels were calculated using the 2^–∆∆Ct^ method, with GAPDH serving as the endogenous control, as relative fold-changes. The following primer sequences were used: *LAMP1*, 5′-cgcttgaatatgactcttcc-3′ (sense) and 5′-aacacactcttccacagacc-3′ (antisense); *LAMP2*, 5′-agcttttgtccaaaatggta-3′ (sense) and 5′-attgccatttctaatgctgt-3′ (antisense); *CTSD*, 5′-gaagctaggaggcaaaaact-3′ (sense) and 5′-cgaccctattgttgtctctg-3′ (antisense); *PGC1*, 5′- agcagaaagcaattgaagag-3′ (sense) and 5′-aggtgtaacggtaggtgatg-3′ (antisense); *MCOLN1*, 5′-ggttactctgatgggtctga-3′ (sense) and 5′-cttggatcaatgtcaaaggt-3′ (antisense), and *GAPDH*, 5′-aacctgccaaatatgatgaca-3′ (sense) and 5′-ataccaggaaatgagcttgac-3′ (antisense).

**Immunocytochemistry**

NCTC clone 1469 cells (5 × 10⁵ cells/well) were seeded in 12-well plates and cultured for 24 h. Cells were then treated with or without palmitate (0.2 mM) for 24 h in the presence or absence of platycodin D (20 μM). For immunocytochemistry, cells were fixed with 4% paraformaldehyde for 10 min and permeabilized with 0.25% Triton X-100 for 10 min at room temperature. Cells were subsequently incubated overnight at 4 °C with the following primary antibodies: anti-LC3 (PM036, MBL), anti-LAMP2 (sc-18822, Santa Cruz), or anti-TFEB (A303673A, Thermo Fisher). After washing with PBS, cells were incubated with Alexa Fluor 488-conjugated (A-11008, Molecular Probes) or Alexa Fluor 594-conjugated (A-11007, Molecular Probes) secondary antibodies for 2 h at room temperature. Nuclei were counterstained with DAPI (D8417, Sigma-Aldrich) for 5 min. A Zeiss LSM 880 confocal microscope (Carl Zeiss, Oberkochen, Germany) was used to acquire images, which were subsequently processed using ZEN 2009 software (version 5.5 SP1; Zeiss). All procedures were performed in triplicate on coverslips.

**BODIPY 493/503 and Lysotracker staining**

NCTC clone 1469 cells (5 × 10⁵ cells/well) were seeded in 12-well plates and cultured for 24 h. Thereafter, the cells were treated with or without palmitate (0.2 mM) for an additional 24 h in the presence or absence of platycodin D (20 μM). For lipid droplet and lysosome staining, cells were washed with 1× PBS and incubated with BODIPY 493/503 (17-1084672, Invitrogen) and LysoTracker™ Red DND-99 (L7528, Invitrogen) in a humidified incubator at 37 °C with 5% CO₂. Imaging was carried out using a Zeiss LSM 880 confocal microscope (Carl Zeiss, Oberkochen, Germany), and image acquisition was managed using ZEN 2009 software (version 5.5 SP1; Zeiss). Triplicate coverslips were used for each experiment.

**Autophagic flux**

NCTC clone 1469 cells (5 × 105 /well) were cultured for 24 h on a coverslip in a 12-well plate for 24 h. Cells were transfected with the GFP-RFP-LC3 plasmid (1 µg) (117413, addgene) using lipofectamine 3000 (Thermo Fisher Scientific). After 24 h, cells were treated with or without palmitate (0.2 mM) for 24 h, and with or without platycodin D (20 μM) for an additional 24 h. The stimulated cells on coverslips were washed three times with PBS and fixed with 4% paraformaldehyde for 10 min. Nuclei were counterstained with DAPI (D8417, Sigma-Aldrich) for 5 min. After sample mounting, fluorescence images were obtained using a Zeiss LSM 880 confocal laser scanning microscope (Carl Zeiss, Oberkochen, Germany). ImageJ software was employed to assess autophagic flux. All slides were examined in triplicate, and a minimum of 200 cells were quantified per well.

**si*TFEP* Transfection into hepatocytes**

NCTC clone 1469 cells were seeded in 12-well plates and cultured to 50%–60% confluence in DMEM supplemented with 10% FBS. For TFEB silencing, cells were transfected with either TFEB siRNA (sc-38509, Santa Cruz) or scrambled control siRNA (sc-37007, Santa Cruz) using Lipofectamine RNAiMAX (13778150, Invitrogen), following the manufacturer’s guidelines.

**Statistical analysis**

All data are expressed as mean ± standard deviation (SD). Statistical significance between two independent groups was assessed using the Mann–Whitney U test. All analyses were conducted using SPSS Statistics version 21.0 (IBM Corp., Armonk, NY, USA) for Windows. A p-value < 0.05 was considered statistically significant.

**Supplementary References**

1. Hunter AK, Lin HC. Review of clinical guidelines in the diagnosis of pediatric nonalcoholic fatty liver disease. *Clin Liver Dis*. 2021;18:40-44. doi.org/10.1002/cld.1094.

2. Younossi ZM. Non-alcoholic fatty liver disease - A global public health perspective. *J Hepatol*. 2019;70:531-544. doi.org/10.1016/j.jhep.2018.10.033.

3. Shan GY, Wan H, Zhang YX, et al. Pathogenesis and research progress of nonalcoholic fatty liver disease/nonalcoholic steatohepatitis. *World J Hepatol*. 2024;16:1142-1150. doi.org/10.4254/wjh.v16.i10.1142.

4. Masuoka HC, Chalasani N. Nonalcoholic fatty liver disease: an emerging threat to obese and diabetic individuals. *Ann N Y Acad Sci*. 2013;1281:106-122. doi.org/10.1111/nyas.12016.

5. Zhang QQ, Lu LG. Nonalcoholic fatty liver disease: dyslipidemia, risk for cardiovascular complications, and treatment strategy. *J Clin Transl Hepatol*. 2015;3:78-84. doi.org/10.14218/JCTH.2014.00037.

6. Martinez-Lopez N, Singh R. Autophagy and lipid droplets in the liver. *Annu Rev* *Nutr*. 2015;35:215-237. doi.org/10.1146/annurev-nutr-071813-105336.

7. Lebeaupin C, Vallee D, Hazari Y, et al. Endoplasmic reticulum stress signalling and the pathogenesis of non-alcoholic fatty liver disease. *J Hepatol*. 2018;69:927-947. doi.org/10.1016/j.jhep.2018.06.008.

8. Carotti S, Aquilano K, Zalfa F, et al. Lipophagy impairment is associated with disease progression in NAFLD. *Front Physiol*. 2020;11:850. doi.org/10.3389/fphys.2020.00850.

9. Kashima J, Shintani-Ishida K, Nakajima M, et al. Immunohistochemical study of the autophagy marker microtubule-associated protein 1 light chain 3 in normal and steatotic human livers. *Hepatol Res*. 2014;44:779-787. doi.org/10.1111/hepr.12183.

10. Fukuo Y, Yamashina S, Sonoue H, et al. Abnormality of autophagic function and cathepsin expression in the liver from patients with non-alcoholic fatty liver disease. *Hepatol Res*. 2014;44:1026-1036. doi.org/10.1111/hepr.12282.

11. Napolitano G, & Ballabio A. TFEB at a glance*. J Cell Sci*. 2016;129:2475-81. doi.org/ 10.1242/jcs.146365.

12. Sardiello M, Palmieri M, di Ronza A, et al. A gene network regulating lysosomal biogenesis and function. *Science*. 2009;325:473-477. doi.org/10.1126/science.1174447.

13. Yu S, Wang Z, Ding L & Yang L, The regulation of TFEB in lipid homeostasis of non-alcoholic fatty liver disease: Molecular mechanism and promising therapeutic targets. *Life Sci*. 2020;246:117418. doi.org/ 10.1016/j.lfs.2020.117418.

14. Zhou W, Yan X, Zhai Y, et al. Phillygenin ameliorates nonalcoholic fatty liver disease viaTFEB-mediated lysosome biogenesis and lipophagy. *Phytomedicine*. 2022;103:154235. doi.org/10.1016/j.phymed.2022.154235.

15. Guan L, Guo L, Zhang H, et al. Naringin protects against non-alcoholic fatty liver disease by promoting autophagic flux and lipophagy. *Mol Nutr Food* *Res*. 2024;68:e2200812. doi.org/10.1002/mnfr.202200812.

16. Zhang H, Lu J, Liu H, et al. Ajugol enhances TFEB-mediated lysosome biogenesis and lipophagy to alleviate non-alcoholic fatty liver disease. *Pharmacol Res*. 2021;174:105964. doi.org/10.1016/j.phrs.2021.105964.

17. Kim MS, Hur YG, Kim WG, et al. Inhibitory effect of Platycodon grandiflorum on T(H)1 and T(H)2 immune responses in a murine model of 2,4-dinitrofluorobenzene-induced atopic dermatitis-like skin lesions. *Ann Allergy Asthma Immunol*. 2011;106:54-61. doi.org/10.1016/j.anai.2010.10.020.

18. Zhang LL, Huang MY, Yang Y, et al. Bioactive platycodins from *Platycodonis* *Radix*: Phytochemistry, pharmacological activities, toxicology and pharmacokinetics. *Food Chem.* 2020;327:127029. doi.org/10.1016/j.foodchem.2020.127029.

19. Zhang J, Song N, Liu Y, et al. Platycodin D inhibits beta-amyloid-induced inflammation and oxidative stress in BV-2 cells via suppressing TLR4/NF-kappa B signaling pathway and activating Nrf2/HO-1 signaling pathway. *Neurochem Res*. 2021;46:638-647. doi.org/10.1007/s11064-020-03198-6.

20. Li W, Tian YH, Liu Y, et al. Platycodin D exerts anti-tumor efficacy in H22 tumor-bearing mice via improving immune function and inducing apoptosis. *J Toxicol Sci*. 2016;41:417-428. doi.org/10.2131/jts.41.417.

21. Cho SY, Song CH, Lee JE, et al. Effects of platycodin D on reflux esophagitis due to modulation of antioxidant defense systems. *Evid Based Complement Alternat Med.* 2018;2018:7918034. doi.org/10.1155/2018/7918034.

22. Shen Q, Zhong YT, Liu XX, et al. Platycodin D ameliorates hyperglycaemia and liver metabolic disturbance in HFD/STZ-induced type 2 diabetic mice. *Food Funct*. 2023;14:74-86. doi.org/10.1039/d2fo03308a.

23. Liu H, Xu L, Lu E, et al. Platycodin D facilitates antiviral immunity through inhibiting cytokine storm via targeting K63-linked TRAF6 ubiquitination. *J Leukoc Biol.* 2025;117:qiae075. doi.org/10.1093/jleuko/qiae075.

24. Hwang YP, Choi JH, Kim HG, et al. Saponins, especially platycodin D, from Platycodon grandiflorum modulate hepatic lipogenesis in high-fat diet-fed rats and high glucose-exposed HepG2 cells. *Toxicol Appl Pharmacol*. 2013;267:174-183. doi.org/10.1016/j.taap.2013.01.001.

25. Tang X, et al. Platycodon D reduces obesity and non-alcoholic fatty liver disease induced by a high-fat diet through inhibiting intestinal fat absorption. *Front Pharmacol.* 2024;15:1412453. doi.org/10.3389/fphar.2024.1412453.

**Supplementary Figure Captions**

**Figure S1. Effect of Platycodin D on palmitate-Induced cytotoxicity in NCTC Clone 1469 cells.** (A-B) NCTC clone 1469 cells were stimulated with or without palmitate (0.2 mM) in the presence and absence of platycodin D (20 μM). The viability of NCTC clone 1469 cells was determined via the CCK-8 assay. Experiments were repeated at least three times. Data are expressed as mean ± SD. ***p* < 0.01. PD, platycodin D.

**Figure S2. Platycodin D alleviated hepatic lipid accumulation by activating lipophagy.** (A-G) NCTC clone 1469 cells were stimulated with or without palmitate (0.2 mM) in the presence and absence of platycodin D (20 μM). (A) Quantification of lipid droplets per cell. (B) Quantification of LC3 punctate foci per cell. (C) Quantification of p62 foci per cell. (D) Quantification of the number of red puncta per cell. (E) Quantification of lipid droplets cell. (F) HepG2 cells were stimulated with or without palmitate (0.2 mM) in the presence and absence of platycodin D (20 μM). HepG2 cells were stained with BODIPY 493/503 in the presence and absence of CQ (20 μM). (G) Quantification of lipid droplets cell. Experiments were repeated at least three times. Data are expressed as mean ± SD. ***p* < 0.01. Scale bar: 10 µm. Veh, vehicle; PA, palmitate; PD, platycodin D; CQ, chloroquine.

**Figure S3. Platycodin D promoted lysosome biogenesis in palmitate-induced hepatocyte cells.** (A-B) NCTC clone 1469 cells were stimulated with or without palmitate (0.2 mM) in the presence and absence of platycodin D (20 μM) (A) Quantification of colocalization of the LC3 puncta with LAMP2 merge per cell. (B) Quantification of a merge of the lipid droplets and lysosome per cell. Experiments were repeated at least three times. Data are expressed as mean ± SD. ***p* < 0.01. Veh, vehicle; PA, palmitate; PD, platycodin D.

**Figure S4. Platycodin D-induced TFEB activation regulated lysosome biogenesis in hepatocytes.** (A-D) NCTC clone 1469 cells were stimulated with or without palmitate (0.2 mM) in the presence and absence of platycodin D (20 μM) (A) Quantification of the nuclear translocation of TFEB localization per cell. (B-D) NCTC clone 1469 cells were transfected with non-silencing control siRNA (control) or siRNA targeting *TFEB* (siTFEB). (B) Quantification of the number of red puncta per cell. (C) Quantification of colocalization of the LC3 puncta with LAMP2 merge per cell. (D) Quantification of a merge of the lipid droplets and lysosome per cell. Experiments were repeated at least three times. Data are expressed as mean ± SD. ***p* < 0.01. Veh, vehicle; PA, palmitate; PD, platycodin D.

**Figure S5. Working model illustrating how platycodin D alleviates hepatic steatosis by promoting TFEB-mediated lysosomal biogenesis, restoring autophagic flux, and enhancing lipophagy.** Platycodin D facilitates the nuclear translocation of TFEB, which upregulates lysosome-associated genes (e.g., LAMP1, LAMP2, ATP6V1A, CTSD, PGC1, and MCOLN1), thereby restoring lysosomal function, promoting lipophagy, and facilitating the clearance of lipid droplets. TFEB deficiency abolishes these effects, indicating that TFEB plays a central role in the lipid-lowering and autophagy-enhancing actions of platycodin D in hepatocytes.
